# A donor-recipient ranking model to optimize long-term survival post liver transplant

**DOI:** 10.1038/s41598-026-52933-6

**Published:** 2026-06-16

**Authors:** Eunice Tan, Yingke Wang, Yingji Sun, Xi He, Sirisha Rambhatla, Mamatha Bhat

**Affiliations:** 1 Department of Medicine, Yong Loo Lin School of Medicine, National University of Singapore, Singapore, Singapore; 2 National University Centre for Organ Transplantation, National University Health System, Singapore, Singapore; 3 National University Centre for Digestive Health, National University Hospital, Singapore, Singapore; 4 University of Waterloo, Waterloo, Canada; 5 Department of Medicine, Ajmera Transplant Center, University Health Network, University of Toronto, Toronto, Canada

**Keywords:** Liver transplantation, Donor-recipient matching, Machine learning, Cancer, Gastroenterology, Medical research, Oncology

## Abstract

Liver transplant (LT) is a life-saving treatment for patients with cirrhosis and/or hepatocellular carcinoma (HCC), but organ shortages and suboptimal donor-recipient matching remain major challenges and existing donor-recipient matching risk models offer limited predictive accuracy. Our study aims to develop a machine-learning-based model to predict and rank long-term post-transplant survival. We included adult deceased donor liver transplant recipients from the Scientific Registry of Transplant Recipients (2009–2019). We developed a first of its kind XGBoost-based survival model to predict graft survival at important discrete timepoints and built a corresponding ranking score. The reliability of the score was compared to existing scores. We included 60,649 waitlist registrants, of which 41,058 (67.7%) were males. We found that our predictive model, *DisCScore*, was the strongest at predicting donor-recipient pair survival times, with a C-index of 0.858 (95%CI: 0.8525–0.8643). We achieved the highest mean time-dependent AUROC at 6-months, 1- and 3- years post-LT using *DisCScore* (mean AUC (95% CI): 0.797 (0.783–0.812), 0.821 (0.809–0.832) and 0.865 (0.857–0.874) respectively and *DisCScore* outperformed CoxPH and DeepSurv models in all three timepoints. We found that our model performed best in the high MELD group (MELD > 30) – where the C-index was the highest: 0.873 (95% CI 0.863–0.891), as compared to the low and medium MELD groups. Our proposed model can accurately predict donor-recipient pair survival probabilities, especially in those recipients with high MELD scores. This has the potential to be integrated into the complex decision-making pathway of organ allocation.

**Supplementary Information:**

The online version contains supplementary material available at https://doi.org/10.1038/s41598-026-52933-6.

## Introduction

Liver transplant (LT) is a curative option for patients with cirrhosis and/or hepatocellular carcinoma (HCC) and has excellent medium-long term survival rates^1^. However, as with other solid organ transplantation, donor organ shortage remains a problem^2^. To circumvent the supply–demand mismatch, marginal livers have increasingly been used to augment the pool of donor organs^2,3^. Yet, most centers presently do not have indices to reliably predict if a donor-recipient pair would result in successful transplant with graft longevity^4^. In recent years, multiple models such as the donor risk index (DRI)^4^, survival outcome following liver transplantation (SOFT)^5^, survival benefit (SB) model, balance of risk (BAR)^6^, D-MELD^7^, improved donor-to-recipient allocation score for deceased donors only (ID2EAL-D) scores^8^ have been developed to supplement urgency-based metrics to also measure transplant utility. However, at present, there remains neither consensus on key donor-recipient factors to be considered nor the optimal risk prediction models to use^9^. While scores such as the DRI, SOFT, BAR, ID2EAL-D may be simple to implement, their static nature and limitations in features often fail to capture complex time-varying interactions and hence have modest reliability^10^. Many of these scores have been not widely adopted due to inherent concerns by the medical community on its reliability, in context of the LT allocation system being one that prioritizes urgency over utility^11,12^. With the use of more marginal organs^4,13^, increased usage of machine perfusion and ongoing policy changes^14^, it is likely that performance of existing models would further decrease with time^4,15^. Deep learning techniques which capture complex non-linear relationships, have been shown to surpass conventional statistical methods in predicting graft survival^16,17^. Yet neural network-based survival models like DeepSurv and DeepHit while allowing non-linear analysis, are prone to overfitting especially if data quality is insufficient^18^. Conversely, Random Survival Forest (RSF) models are well-suited to heterogenous clinical datasets, as tree-based models can inherently handle missing values by treating them as a separate category during node splitting^19,20^. They simplify the data preparation process by eliminating the need for explicit imputation and capture non-linear and interaction effect among donor and recipient attributes. Given these known limitations, the objective of our study was to predict survival probabilities of each donor-recipient pair at 6-months, 1-year and 3-years post-transplant and thereby guiding personalization of an optimal donor profile for each individual recipient. We calculate a donor-recipient compatibility score (DisCScore) for each donor-recipient pair that predicts aggregated score of outcomes at these discrete time points. We compare the performance of our compatibility score with existing clinical scores. Lastly, we provide model interpretability by reporting variable importance to improve transparency to clinicians.

## Methods

### Data selection and study population

Our study utilized retrospective data from the Scientific Registry of Transplant Recipients (SRTR) which includes data on all donors, waitlisted candidates, and transplant recipients in the U.S., submitted by the members of the Organ Procurement and Transplantation Network (OPTN). Briefly, the SRTR/UNOS database administers the OPTN under contract with the U.S. Department of Health and Human Services. The U.S. transplant system operates under strict federal ethical and legal standards that prohibit procurement of organs from prisoners for transplantation research. This deidentified database contains individual level available data upon request on all waitlist registrants and transplant recipients undergoing listing for solid organ transplantation in the US since September 1985. The study population includes only deceased donor liver transplant (DDLT) patients, from January 2009 to December 2019. We included all patients aged > 18 years of age who underwent deceased donor LT. We excluded pediatric transplants, living donor LT and multi-organ transplants. We excluded patients waitlisted with Status 1 as those were patients who required urgent transplantation. Inclusion and exclusion criteria for all patients used in this analysis are provided in Supplementary Fig. 1. The study was conducted in accordance with the Declaration of Helsinki. The requirement for informed consent was waived by (University Health Network Research Ethics Board Study #21–5783) as the study used de-identified registry data and no identifiable patient information was accessed.

### Data pre-processing and feature selection

We first applied he data-cleaning framework of Berrevoets et al. to identify clinically meaningful predictors while minimizing target leakage in the liver transplant context^21^. As Registry data (e.g., SRTR/UNOS) are heterogeneous in quality and completeness across centers, it can degrade model performance if features are chosen solely by expert opinion. To address this, we used a two-stage, data-driven procedure that combines model-guided salience with statistical evidence. We refined the candidate set through sequential statistical screening: variables with > 20% missingness or with zero/near-zero variability across the cohort were excluded. The remaining features underwent clinician adjudication to confirm clinical relevance and remove contextually irrelevant or non-actionable variables.

We first trained the initial model to differentiate two groups of data instances: (i) Group 1, pairs with favorable long-term graft survival and high predicted rank; and (ii) Group 2, pairs with high predicted rank but comparatively poor survival. By comparing these two groups, we identify the features the model relies upon when distinguishing strong from weak donor-recipient matches (Stage 1). In Stage 2, The feature set is further refined using statistical tests. Continuous variables were compared using two-sample t-tests and categorical variables were compared using χ^2^ tests. Features meeting statistical significance were flagged as survival relevant. Finally, the final feature set comprises of the union of (a) statistically significant features from Stage 2 and (b) the top 70 model-important features from Stage 1. This integration ensures that retained variables are supported both by model salience and by between-group differences in observed outcomes (Supplementary Fig. 2).

### Model development and assessment

We stratified the unified dataset into bins of graft-survival time so to ensure that they are proportionally represented in both the training (80%) and validation (20%) set. The training subset was further split into training (95%) and validation (5%) for hyper-parameter optimization. To eliminate and minimize the possibility of obtaining results due to randomness, we performed the train-test split using 10 different random seeds and aggregated the performance results.

**Fig. 1:**
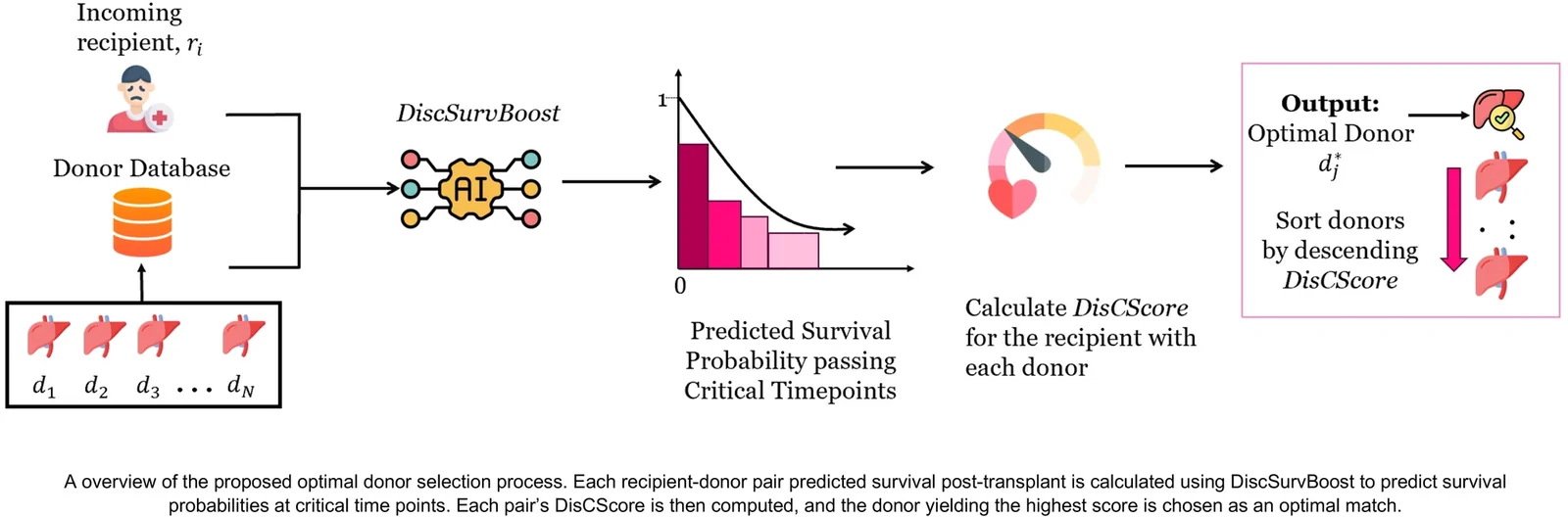
An overview of the proposed optimal donor selection process.

Existing deep learning approaches typically predict patient survival (time-to-event or risk) on a continuous scale. However, this task is complicated by data limitations and insufficient availability of essential features. For instance, certain important variables such as "deceased donor-dopamine" and "deceased donor-dopamine within 24 h pre-cross clamp" are no longer collected. additionally, crucial features like “primary diagnosis” remain highly complex, comprising over 15 distinct. Given these constraints, directly predicting exact survival times requires models with very high predictive power and comprehensive data, making it particularly challenging. To mitigate these issues, we adopt a more flexible, discrete-time approach by predicting survival probabilities at predefined intervals. By modeling survival at multiple discrete time points as separate prediction tasks, the model estimates probabilities of event occurrence within specific intervals. This multi-label strategy simplifies the prediction process by concentrating on the likelihood of survival past clinically relevant milestones rather than identifying precise event times. Furthermore, it aligns closely with clinical practices, where patient outcomes are routinely assessed at fixed checkpoints such as three months, six months, one year, and beyond (Fig. 1). We assess the discriminative capability of the DisCScore, which identifies the most suitable donor for a given patient by integrating discrete time survival probabilities at 6-months, 1-years and 3-years, to known clinical scores such as DMELD, BAR, traditional statistical models such as Cox Proportional Hazard (CoxPH) and RSF, as well as DeepSurv and DeepHit. Every comparator was retrained on the identical training folds and tuned with grid or Bayesian search, ensuring a genuinely head-to-head evaluation. Global discrimination was quantified with Harrell’s concordance index (C-index), timed-receiver operating characteristic (timed-ROC) and timed-area under the curve (timed-AUC) at clinically relevant timepoints of 6-months, 1-year and 3-year. For each metric, we also reported 95% confidence intervals (CI) estimated by patient-level bootstrap resampling of the fixed test cohort with 1000 replicates. In each replicate, patients were sampled with replacement from the original test cohort to form a bootstrap sample of equal size to the original test set, and the corresponding metric was recalculated. The 95% CI was then defined by the 2.5^th^ and 97.5^th^ percentiles of the empirical bootstrap distribution. For allocation simulations, we compared the C-index between optimal versus suboptimal donor-recipient pairings. To provide interpretability, we present the top 20 most frequently selected features of the model. This is performed by applying the above compatibility score to rank donors for each waitlist candidate. Lastly, we perform sensitivity analysis over important groups of patients, such as those with low (< 20), average (20–29), high (> 30) MELD subgroups, as well as those with and without HCC.

## Results

### Study population

We included 60,649 waitlist registrants, of whom 41,058 (67.7%) registrants were males and 19,591 (32.3%) females. The mean ± SD age of the patients was 57 ± 12 years with a median (IQR) MELD of 21 (13–30) at time of waitlisting. Most of the waitlist registrants required LT due to hepatitis C cirrhosis followed by alcohol associated liver cirrhosis and metabolic dysfunction associated steatohepatitis. A third (33%) of patients were waitlisted for LT due to HCC. Median (IQR) follow-up time was 5.95 (3.76–8.96) years. 92% and 85% patients survived past 1- and 3-years post-LT. The median (IQR) age of donor was 42 (28–55) years and more than half (59.8%) were males. The full characteristics of waitlist registrants and their donors are presented in Table 1,2.

**Table 1:** Recipient characteristics of patients included in the study.

	**Waitlisted patients** **(n = 60,649)**	**Training Data** **(n = 48,519)**	**Test Data** **(n = 12,130)**
**Demographics at registration**			
Age, years	57.0 (51–63)	57 (51 -63)	57 (51 -63)
Gender, male (%)	41,058 (67.7)	32,866 (67.74)	8192 (67.54)
BMI	28.1(24.4–32.3)	28.1 (24.5—32.3)	28.08 (24.56—32.28)
Ethnicity			
*White, non-hispanic*	34,675(71.5)	34,675 (74.5)	8724 (71.92)
*Black, non-Hispanic*	4189.0 (8.63)	4189 (8.6)	1023 (8.43)
*Asian, non-hispanic*	2071.0 (4.3)	2071 (4.3)	520 (4.29)
*Hispanic*	6938.0 (14.3)	6938 (14.3)	1664 (13.72)
*American Indian*	430 (0.71)	97 (0.8)	333 (0.69)
**Etiology of liver disease**			
Hepatitis C(%)	13,740 (22.65)	10,937 (22.5)	2803 (23.11)
MASH(%)	7753 (12.78)	6205 (12.8)	1548 (12.76)
Alcohol associated liver disease(%)	14,185 (23.39)	11,421 (23.5)	2764 (22.79)
PBC(%)	1589 (2.62)	1278 (2.6)	311 (2.56)
PSC(%)	2679 (4.42)	2131 (4.4)	548 (4.52)
Others*(%)	7102 (11.72)	6545 (11.8)	5698 (11.74)
HCC at time of listing (%)	8136 (13.41)	6484 (13.4)	1404 (59.82)
**Clinical characteristics at transplant**			
MELD	21 (13–30)	21 (13–30)	21 (13–30)
Sodium (mmol/L)	136 (133–139)	136 (133 – 139)	136 (133.00—139.00)
Bilirubin (mg/dL)	3.80 (9.20)	3.8 (1.70–10.90)	3.8 (1.70—11.00)
Albumin (g/dL)	3.10 (1.00)	3.1 (2.60 – 3.60)	3.1 (2.60—3.60)
INR	1.60 (0.90)	1.6 (1.30 – 2.20)	1.6 (1.30—2.20)
Creatinine (mg/dL)	1.04(0.75)	1.04 (0.80—1.56)	1.03 (0.80—1.52)
Dialysis at transplant (%)	4868 (10.05)	4868 (10.05)	1206 (9.96)
Ascites (%)	44,538 (73.44)	35,698 (73.58)	8840 (72.88)
Portal vein thrombosis (%)	8088 (13.34)	6571 (13.54)	1517 (12.51)

*Others: include autoimmune hepatitis, cryptogenic cirrhosis, hepatitis B cirrhosis, alpha-1-antitrypsin deficiency, cholangiocarcinoma, hemochromatosis, and polycystic liver disease. Continuous variables expressed in mean (SD) or median (range). Abbreviations: BMI, body mass index; MASH, metabolic dysfunction assoiciated steatohepatitis; PBC, primary biliary cholangitis; PSC, primary sclerosing cholangitis; HCC, hepatocellular carcinoma; INR, international normalized ratio.

**Table 2:** Donor characteristics at registration of patients included in the study.

	**Waitlisted patients** **(n = 60,649)**	**Training Data** **(n = 48,519)**	**Test Data** **(n = 12,130)**
Age, years (range)	42 (28.00—55.00)	42 (28.00—55.00)	42 (28.00—55.00)
Gender, male (%)	36,238 (59.75)	28,934 (59.63)	7304 (60.21)
Ethnicity(%)			
*White, non-hispanic*	39,390 (64.95)	31,569 (65.07)	7821(64.48)
*Black, non-Hispanic*	10,937 (18.03)	8697 (17.92)	2240 (18.47)
*Asian, non-hispanic*	1475 (2.43)	1180 (2.43)	295 (2.43)
*Hispanic*	8288(13.67)	6641 (13.69)	1647 (13.58)
*American Indian*	252 (0.42)	194 (0.4)	58 (0.48)

### Model performance: discriminatory power of ranking score

We found that our predictive score, DisCScore, was the strongest at predicting donor-recipient pair survival times, with a C-index of 0.8584 (95% CI: 0.8525–0.8643). This performance surpassed that of the best-performing baseline model, *DeepHit*, which had a C-index of 0.8477 (95% CI: 0.8411- 0.8541). It also surpassed conventional existing scores such as DMELD and BAR score (C-index: 0.5149 and 0.5237) respectively (Table 3).

**Table 3:** Results from evaluation metrics of existing scores compared to DisCScore.

Model	C-index	95% CI
DMELD	0.515	0.506–0.526
BAR	0.524	0.514–0.534
CoxPH	0.841	0.843–0.848
DeepSurv	0.848	0.841–0.854
DeepHit	0.848	0.841–0.854
DisCScore	0.858	0.853–0.864

Abbreviations: D-MELD: Donor-Model for end-stage liver disease; BAR: balance of risk.

### Model performance at distinct timepoints at 6-months, 1-, 3-year post transplant

**Fig. 2:**
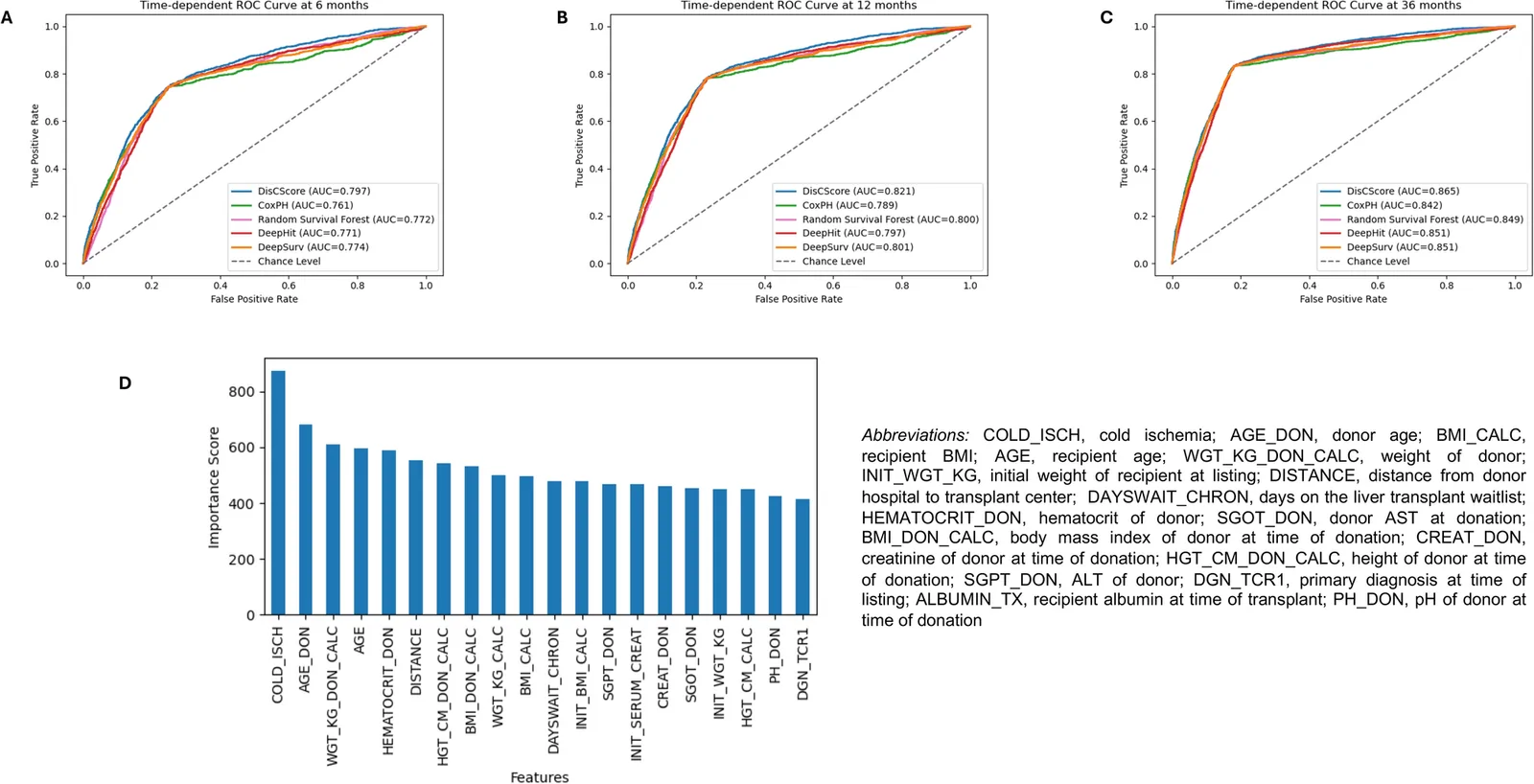
Time-dependent ROC at distinct timepoints at (**a**) 6-months, (**b**) 1- and (**c**) 3- years post-transplant (**d**) Top 20 most important variables for model.

To evaluate and compare the predictive performance of survival models at specific time points, we analyzed the time-dependent AUC at 6 months, 1- and 3-year post-LT periods. We achieved the highest mean time-dependent AUC at 6-months, 1- and 3- years post-LT using *DisCScore* (mean AUC (95% CI): 0.7967 (0.783–0.812), 0.821 (0.809–0.832) and 0.865 (0.857–0.874) respectively. *DisCScore* outperformed CoxPH and RSF, DeepHit and DeepSurv models in all three timepoints (Figs. 2a-c).

### Variable importance

We found the top 20 most frequently selected features in the decision tree, which reflects the variable importance to be mostly in line with those expected clinically to predict recipient survival post-transplant (Fig. 2d). The top ten features that were important to our model included donor variables such as age, weight and hematocrit of donor; recipient variables such as BMI, age, serum creatinine, number of days on waitlist; and transplant variables including cold ischemia time, distance from donor hospital to transplant center.

### Sensitivity analysis of model accuracy across different MELD groups

**Fig. 3:**
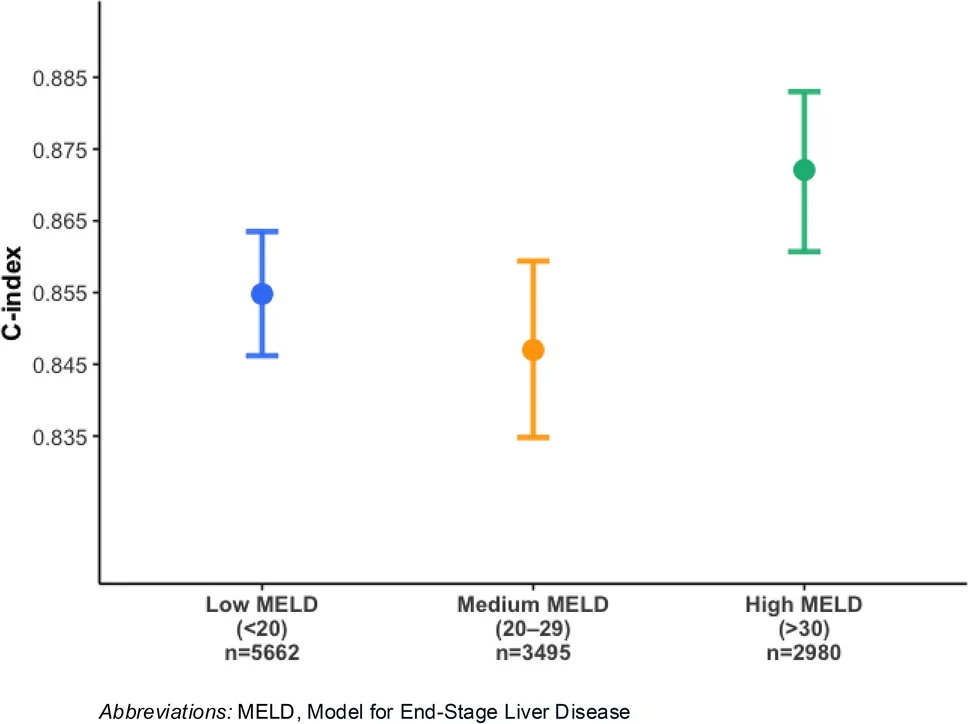
Accuracy of *DisCScore* across different MELD subgroups.

We divided our recipients according to MELD groups, where in low MELD was < 20, medium MELD 20–30 and high MELD > 30. We found that our model performed best in the high MELD group (MELD score > 30), where in the C-index was the highest 0.8721 (95% CI 0.861–0.883), as compared to 0.8548 (95% CI 0.846–0.864) and 0.8470 (95% CI 0.835–0.859) in the low and medium MELD group respectively (Fig. 3).

### Sensitivity analysis of model accuracy across HCC vs non-HCC

We performed sensitivity analysis of model accuracy across patients with HCC vs patients without HCC. We found that model showed higher accuracy in the HCC group: 0.8771 (95% CI 0.8629–0.8912) and 0.8527 (95% CI 0.8460–0.8594) in the non-HCC compared to HCC respectively.

## Discussion

Our study represents a novel machine learning based prediction model using objective measures to optimize donor-recipient matching and predicting post-transplant survival using one of the largest datasets including 60,649 waitlist registrants and deceased organ donor pairs. Our model, the DisCScore, is more reliable compared to existing models, such as the DRI, BAR, D-MELD and SOFT scores. These scores have been built using multivariate models and are static, wherein addition of new data points would not be able to optimize reliability of the score further. More importantly, these existing scores also do not address the reliability of the prediction across important subgroups, namely the sicker candidates, with higher MELD scores and registrants waitlisted for HCC.

In our study, we used a tree-based survival approach, enabling us to capture both non-linear and interaction effects among the donor and recipient attributes and offers interpretability. Our model is superior to conventional regression analysis where the drawback is the difficulty in selecting an appropriate function capable for capturing non-linear relationships. Furthermore, unlike in DeepHit and DeepSurv, which employ deep neural networks to predict time-to-event on a continuous scale^18,22^, we predicted outcomes and clinically important discrete timepoints, and was able to improve overall accuracy of the model compared to DeepHit / DeepSurv. Our model also provides interpretability, where in the top ten variable importance also aligned with that expected from a clinical front. Moreover, of clinical significance is that we found that our prediction model performed best in the high MELD cohort, where decisions for optimal donors are most crucial clinically. Our model also remains reliable for HCC patients as compared to non-HCC patients.

The liver transplant waitlist allocation is one that is based on urgency, due to the nature of liver failure, unlike in other solid organ transplants like kidney transplant. However, in a resource limited situation, it would be necessary optimize donor-recipient survival to make best use of the new donor organ. Many studies have investigated the use of machine learning methods to optimize liver transplant allocation, and an additional dimension to the enhancing equity in liver transplant allocation would be to integrate transplant utility, to ensure best use of the donor organ. Our model provides clinicians absolute survival probabilities at important discrete timepoints, that can be communicated to patients more intuitively than abstract indices or that provided as a general statistic.

While we demonstrate that our method is more discriminative than existing approaches, our study is not without limitations. First, our model predominantly focuses on post-transplant survival outcomes but does not consider the risk of waitlist dropout while waiting for an optimal donor. Current allocation systems prioritize urgency, and the introduction of utility-based models raises important ethical considerations, particularly the risk of disadvantaging patients with higher medical acuity or less favorable prognostic profiles. In clinical practice, allocation decisions require balancing both waitlist mortality risk and post-transplant survival benefit. As such, a model that optimizes donor–recipient matching without incorporating waitlist dynamics may have limited applicability in isolation. In this regard, the DisCScore is not intended to replace existing allocation frameworks, but rather to complement them by providing individualized estimates of post-transplant outcomes to support more informed donor–recipient matching when multiple options are available. Future work integrating dynamic waitlist risk prediction with post-transplant survival models may provide a more comprehensive and clinically aligned framework for organ allocation. Second, while our study cohort was large, this is based upon retrospective data from a national database which may lack granularity, and our findings require external validation to further strengthen the generalizability of the model. Most of the liver donor-recipient matching has been shown to be more predictive for short-term rather than long-term outcomes. In our study, even though our model accuracy continued to increase in time on follow-up, an inherent limitation is the addition of subsequent post-transplant exposures, such as compliance of medications or control of cardiometabolic risk factors, and these are not being accounted for in this model. Moreover, the absence of more granular phenotypic, molecular, or imaging-derived features, particularly those reflecting tumor biology in HCC, remains a limitation and were not considered due to paucity of information in this retrospective registry. Third, our model is based upon the UNOS dataset which is heavily predominant in deceased donor liver transplants, and its applicability to living donor liver transplants is unclear at present. Especially as living donation becomes increasingly common, the potential utility of our model needs to be evaluated with a larger population, where donor selection is possible. In high volume centers where living donation is predominant, such donor-recipient matching tools may even allow paired exchanges to best optimize post-transplant outcomes.

In summary, we show in our study that machine learning algorithms can help with donor-recipient pairings to optimize post-transplant outcomes. While the liver transplant allocation is predominantly still one based on urgency, our study suggests that post-transplant survival may be improved with optimal donor-recipient matching. This has clinical implications potentially in larger centers where several donors may be available at a single timepoint. Future studies can also leverage on longitudinal data for patients on the waitlist to yield more accurate and dynamic predictions, and inclusion of more living donor liver transplants to improve applicability to this growing group of patients, where this model may be best utilized.

## Supplementary Information


Supplementary Information.


## Data Availability

The data that support the findings of this study are available from the Scientific Registry of Transplant Recipients (SRTR). Restrictions apply to the availability of these data, which were used under license for the current study and are not publicly available. Information regarding data requests can be found at the SRTR Data Requests website. This deidentified data used contains individual level available data upon request on all waitlist registrants and transplant recipients undergoing listing for solid organ transplantation in the US since September 1985.

## References

[1] Serrano, M. T. et al. Mortality and causes of death after liver transplantation: Analysis of sex differences in a large nationwide cohort. *Transpl. Int.* **35**, 10263 (2022). 35615539 10.3389/ti.2022.10263 PMC9124758

[2] Goldaracena, N., Cullen, J. M., Kim, D.-S., Ekser, B. & Halazun, K. J. Expanding the donor pool for liver transplantation with marginal donors. *Int. J. Surg.* **82S**, 30–35 (2020). 32422385 10.1016/j.ijsu.2020.05.024

[3] Xiang, Z. et al. Current understanding of marginal grafts in liver transplantation. *Aging Dis.* **16**, 1036–1058 (2024). 38607739 10.14336/AD.2024.0214 PMC11964436

[4] Flores, A. & Asrani, S. K. The donor risk index: A decade of experience. *Liver. Transpl.* **23**, 1216–1225 (2017). 28590542 10.1002/lt.24799

[5] Rana, A. et al. Survival outcomes following liver transplantation (SOFT) score: a novel method to predict patient survival following liver transplantation. *Am. J. Transplant* **8**, 2537–2546 (2008). 18945283 10.1111/j.1600-6143.2008.02400.x

[6] Dutkowski, P. *et al.* Are there better guidelines for allocation in liver transplantation? A novel score targeting justice and utility in the model for end-stage liver disease era. *Ann. Surg.* **254**, 745–53; discussion 753 (2011). 10.1097/SLA.0b013e3182365081 22042468

[7] Halldorson, J. B., Bakthavatsalam, R., Fix, O., Reyes, J. D. & Perkins, J. D. D-MELD, a simple predictor of post liver transplant mortality for optimization of donor/recipient matching. *Am. J. Transplant.* **9**, 318–326 (2009). 19120079 10.1111/j.1600-6143.2008.02491.x

[8] Asrani, S. K. et al. Assessment of donor quality and risk of graft failure after liver transplantation: The ID2 EAL score. *Am. J. Transplant.* **22**, 2921–2930 (2022). 36053559 10.1111/ajt.17191

[9] *Decision Making in Liver Transplantation--Limited Application of the Liver Donor Risk Index (LDRI)*. 10.1002/lt.23879 PMC4072766 24692309

[10] Mataya, L., Aronsohn, A., Thistlethwaite, J. R. Jr. & Friedman Ross, L. Decision making in liver transplantation--Limited application of the liver donor risk index. *Liver Transpl.* **20**, 831–837 (2014). 24692309 10.1002/lt.23879 PMC4072766

[11] Briceño, J., Calleja, R. & Hervás, C. Artificial intelligence and liver transplantation: Looking for the best donor-recipient pairing. *Hepatobiliary. Pancreat. Dis. Int.* **21**, 347–353 (2022). 35321836 10.1016/j.hbpd.2022.03.001

[12] Guijo-Rubio, D. et al. Statistical methods versus machine learning techniques for donor-recipient matching in liver transplantation. *PLoS ONE* **16**, e0252068 (2021). 34019601 10.1371/journal.pone.0252068 PMC8139468

[13] Lim, W. H. et al. Natural history of NASH cirrhosis in liver transplant waitlist registrants. *J. Hepatol.* **79**, 1015–1024 (2023). 37307997 10.1016/j.jhep.2023.05.034

[14] Matevish, L. E., Vagefi, P. A. & Patel, M. S. Trends in current use of machine perfusion for donation after cardiac death donors in the US. *J. Hepatol.* **81**, e187–e188 (2024). 38679068 10.1016/j.jhep.2024.04.018

[15] Shaffer, L., Abu-Gazala, S., Schaubel, D. E., Abt, P. & Mahmud, N. Performance of risk prediction models for post-liver transplant patient and graft survival over time. *Liver Transpl.* **30**, 689–698 (2024). 38265295 10.1097/LVT.0000000000000326 PMC11175754

[16] Briceño, J. et al. Use of artificial intelligence as an innovative donor-recipient matching model for liver transplantation: Results from a multicenter Spanish study. *J. Hepatol.* **61**, 1020–1028 (2014). 24905493 10.1016/j.jhep.2014.05.039

[17] Reddy, M. S., Varghese, J., Venkataraman, J. & Rela, M. Matching donor to recipient in liver transplantation: Relevance in clinical practice. *World J. Hepatol.* **5**, 603–611 (2013). 24303088 10.4254/wjh.v5.i11.603 PMC3847943

[18] Katzman, J. L. *et al.* DeepSurv: personalized treatment recommender system using a Cox proportional hazards deep neural network. *BMC Med. Res. Methodol.* **18**, (2018). 10.1186/s12874-018-0482-1 PMC5828433 29482517

[19] Ishwaran, H. et al. Random survival forests for competing risks. *Biostatistics* **15**, 757–773 (2014). 24728979 10.1093/biostatistics/kxu010 PMC4173102

[20] Khosravi, P., Vergari, A., Choi, Y., Liang, Y. & Van den Broeck, G. Handling missing data in decision trees: A probabilistic approach. *arXiv [cs.LG]* (2020).

[21] Berrevoets, J. et al. Learning queueing policies for organ transplantation allocation using interpretable counterfactual survival analysis. *ICML* **139**(792), 802 (2021).

[22] Lee, C., Zame, W., Yoon, J. & Van der Schaar, M. DeepHit: A deep learning approach to survival analysis with competing risks. *Proc. Conf. AAAI Artif. Intell.* **32**, (2018).

