# Supplementary material for: A donor-recipient ranking model to optimize long-term survival post liver transplant
Source: Sci Rep. 2026 Jun 16;16:27485. doi: 10.1038/s41598-026-52933-6 (PMC13534588; doi:10.1038/s41598-026-52933-6)
Supplement: Supplementary file 1 [file 41598_2026_52933_MOESM1_ESM.docx]

**Supplementary Figure 1:** Inclusion and exclusion criteria of the study

**Supplementary Figure 2:** Summary of data preprocessing and feature selection
